# Supplementary material for: Steam Activation of Acid-Chars for Enhanced Textural Properties and Pharmaceuticals Removal
Source: Nanomaterials (Basel). 2022 Oct 5;12(19):3480. doi: 10.3390/nano12193480 (PMC9565870; doi:10.3390/nano12193480)
Supplement: Supplementary file 1 [file nanomaterials-12-03480-s001.zip › nanomaterials-1929649-supplementary.pdf]

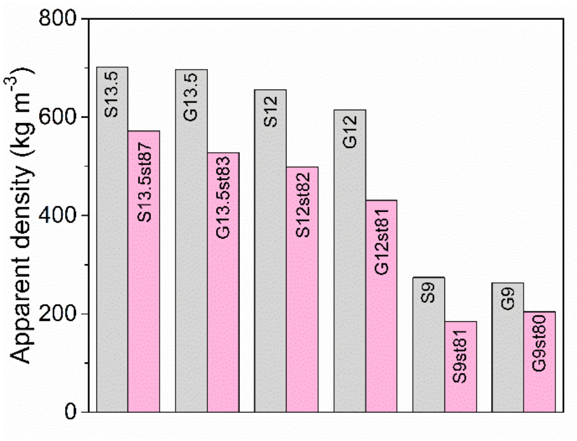

Figure S1. Effect of activation process on apparent density of obtained acid char and AC samples.

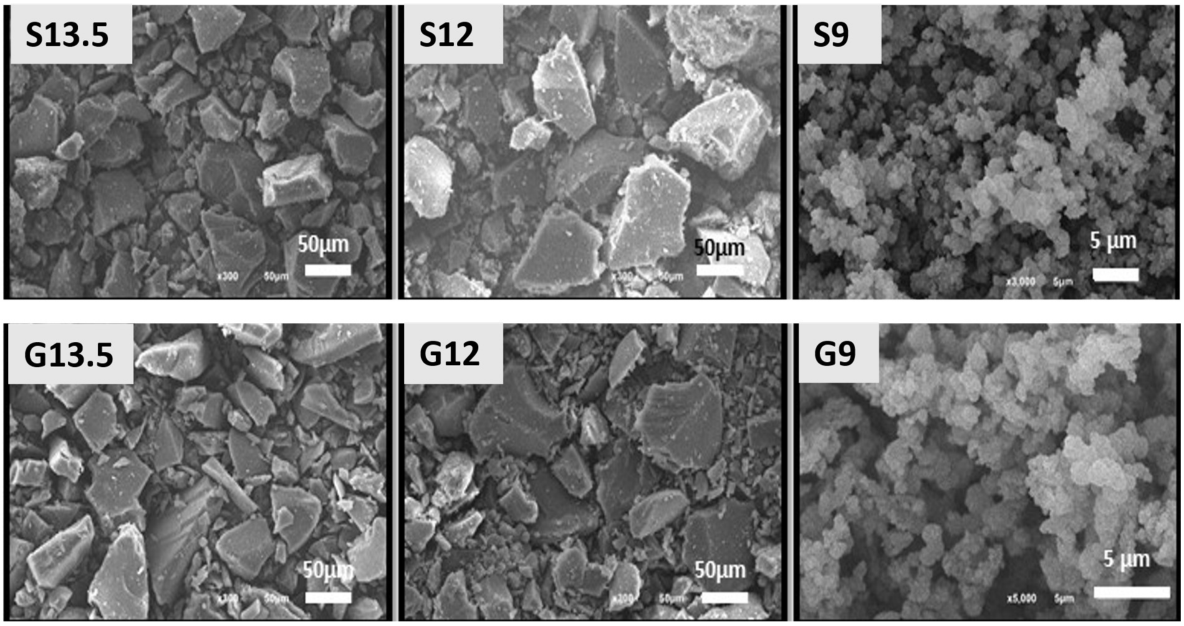

Figure S2. SEM images of acid-char samples obtained by sisal and glucose precursors.

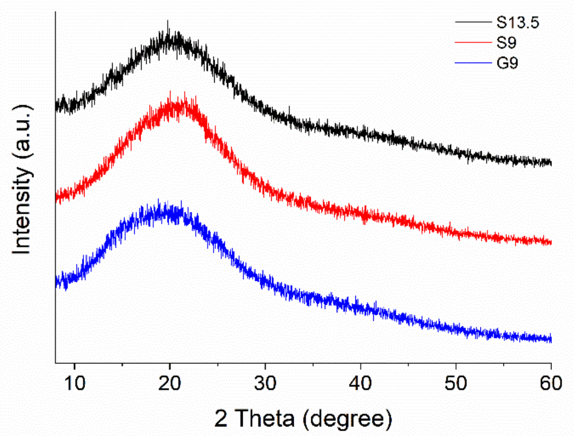

Figure S3. Powder XRD of selected acid-char materials.

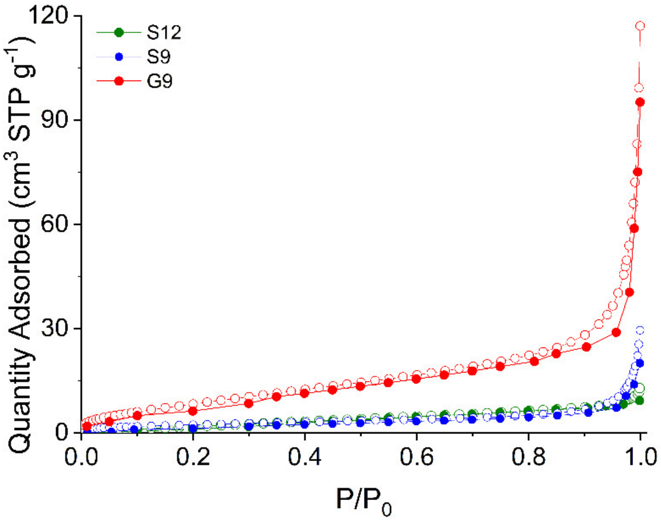

Figure S4. N_2 adsorption-desorption isotherms of sisal- and glucose-based acid-chars.

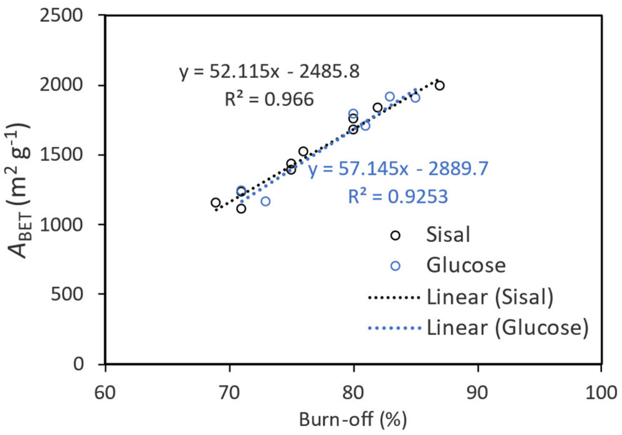

Figure S5. Linear relation between BET area values and burn-off degree during the acid-char steam activation.

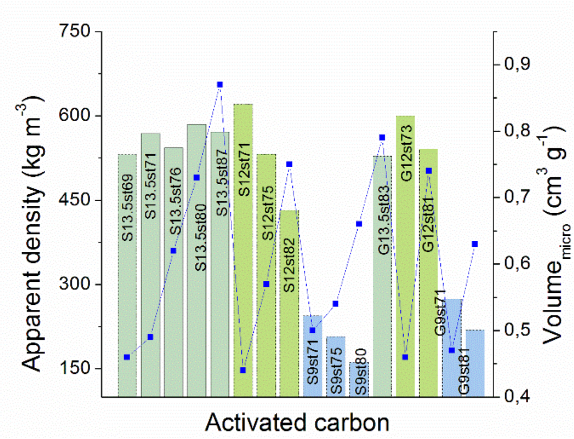

Figure S6. Relation between apparent density and micropore volume of the activated carbons.
